# Treatment of Infected Pseudoarthrosis in a Subtrochanteric Fracture in a Patient with Osteopetrosis

**DOI:** 10.1155/2020/5630202

**Published:** 2020-05-04

**Authors:** Llano Lionel, Jorge D. Barla, Campelo Diego, Taype Danilo, Carlos F. Sancineto, Guido Carabelli

**Affiliations:** Instituto de Ortopedia y Traumatología “Carlos E. Ottolenghi”, Hospital Italiano de Buenos Aires, Argentina

## Abstract

Osteopetrosis is a disease of osteoclasts that results in failure of bone remodeling. Despite the sclerotic radiographic appearance of the thickened cortices and its material hardness, osteopetrotic bone is weak and prone to fracture by minor trauma. We report a case of a subtrochanteric fracture in an osteopetrotic patient, with further pseudoarthrosis and infection. Several surgical procedures were required, with further complications. The outcome of each procedure and the final result are also described.

## 1. Introduction

Osteopetrosis was first described by Albers-Schoenberg when he marked the increased radiographic density of the bones in 1904 [1]. Despite the sclerotic radiographic appearance of the thickened cortices and its material hardness, osteopetrotic bone is weak and prone to fracture by minor trauma. Areas of concentrated stress such as the femoral neck and subtrochanteric areas are especially susceptible [2, 3]. The internal fixation is difficult to perform due to bone fragility; therefore, orthopedists may prefer to use conservative treatment with skeletal traction or plaster application [4]. There is paucity of literature comparing the effectiveness of the conservative versus operative treatment of fractures of long bones in patients with osteopetrosis. Furthermore, the main complications reported in the operative treatment of fractures in patients with osteopetrosis are pseudoarthrosis and osteomyelitis [1, 4]. In the present report, we will describe the surgical management of a patient with an infected pseudoarthrosis associated with a subtrochanteric fracture.

## 2. Case Report

**Figure 1:**
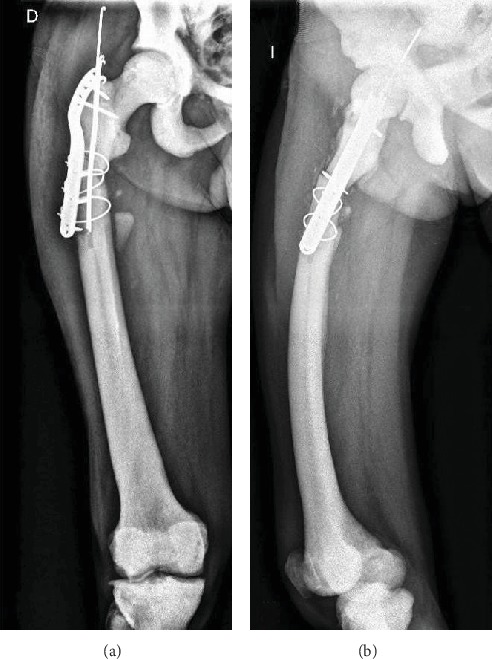
Anteroposterior (a) and lateral (b) radiographs of the femur showing the failure of osteosynthesis of the subtrochanteric fracture.

**Figure 2:**
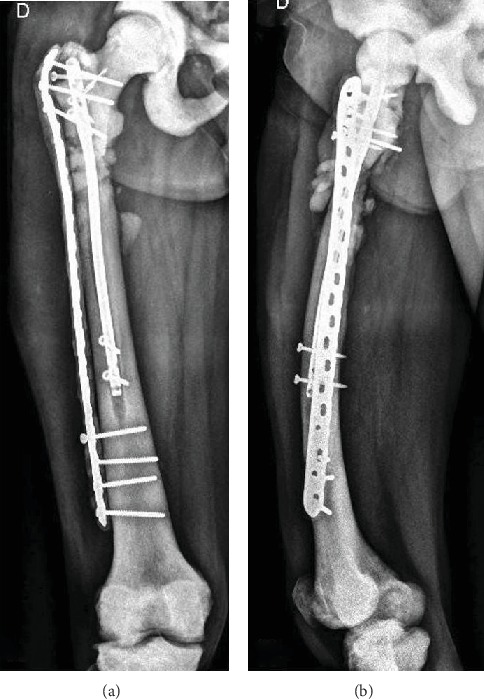
Postoperative anteroposterior (a) and lateral (b) radiographs of the first revision surgery described.

A 64-year-old patient with the diagnosis of osteopetrosis came to external consult with right hip pain and limping. He presented with subtrochanteric fracture in the year 2013. Radiographic images evidenced a failure of osteosynthesis and pseudoarthrosis of the fracture (Figure 1). A biopsy is performed, and the culture result is an Enterococcus Cloacae, sensitive to vancomycin. Surgery is decided and the previous material is removed. We use a long plate in the lateral aspect of the femur (LCP) and an intramedullary nail Expert (Depuy Synthes). Both implants are covered with polymethyl methacrylate cement (PMMA) with local vancomycin (Figure 2).

**Figure 3:**
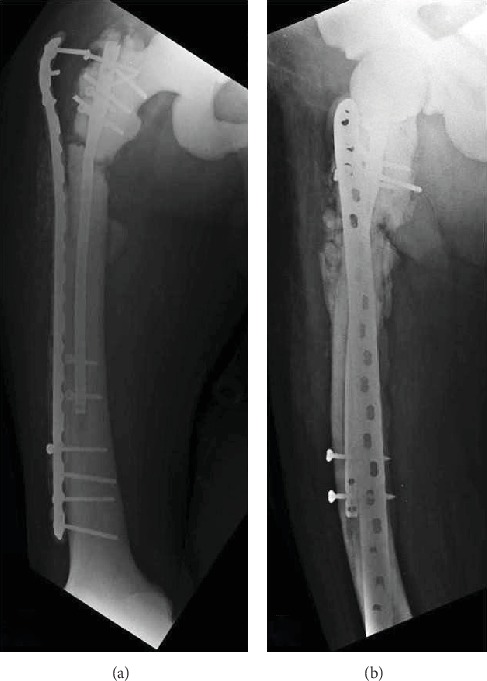
Anteroposterior (a) and lateral (b) radiographs of the femur showing the failure of the first revision configuration.

After 10 months of follow-up, the patient presents with relapse of the pain and limping again. Radiographs evidence a new failure of the material previously used and no consolidation of the previous fracture. A new biopsy is performed with negative results (Figure 3).

**Figure 4:**
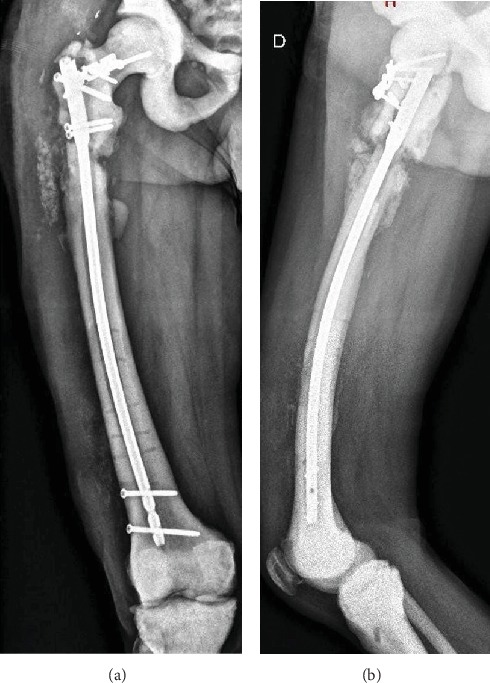
Postoperative anteroposterior (a) and lateral (b) radiographs of the third revision surgery described.

A second revision surgery is done, previous implants are retired, and a femoral neck fracture is evidenced during fluoroscopy. We decided to use a long intramedullary nail (LFN, Depuy Synthes) covered with PMAA and vancomycin (considering previous cultures) and bone allograft for the pseudoarthrosis. Also a reconstruction plate of 3.5 mm is used for the femoral neck fracture, applying compression on the focus (Figure 4).

**Figure 5:**
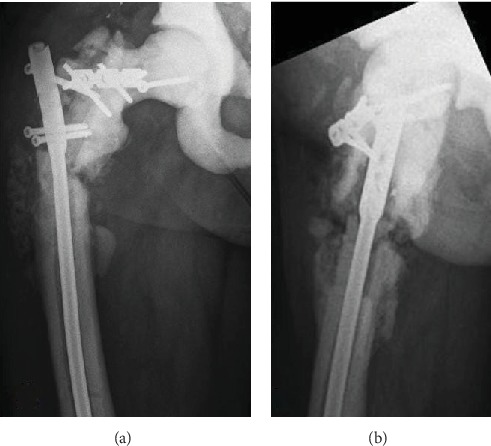
Anteroposterior (a) and lateral (b) radiographs showing the pull out of the intramedullary nail of the femur.

After 6 months of follow-up, in a new radiographic control, we observed a pull out of the nail previously used. Biopsy cultures remain negative, with no reactivation of the previous infection (Figure 5).

**Figure 6:**
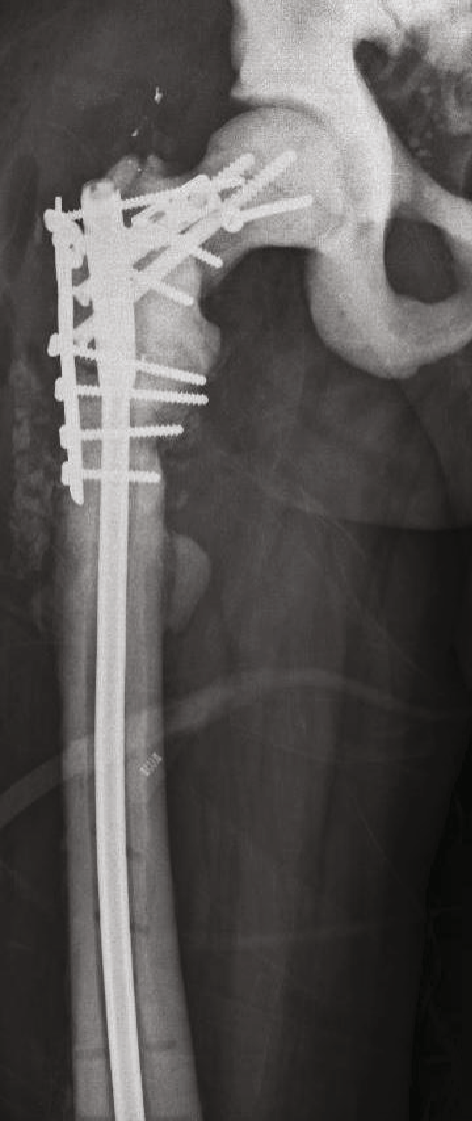
Postoperative anteroposterior radiograph of the femur after the last revision surgery.

A final procedure is performed; we proceeded with new nailing and 2 blocking screws to the femoral neck. Also a T plate is used to gain lateral stability. The femoral neck fracture is consolidated, and no new surgeries were needed (Figure 6).

**Figure 7:**
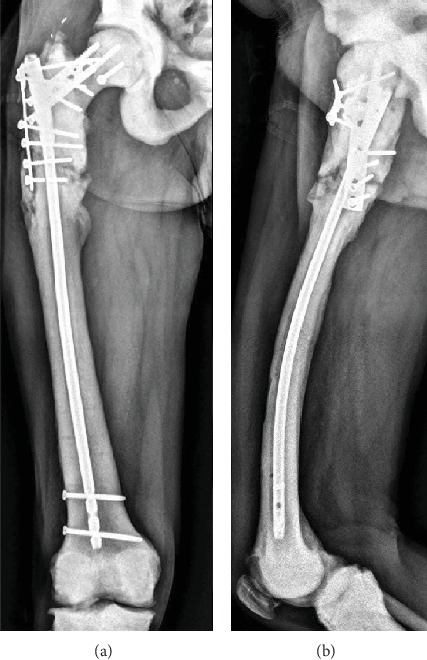
Two-year follow-up. Anteroposterior (a) and lateral (b) radiographs of the femur.

After 2 years of follow-up, the patient has no new complications; he is painless and with no limping. New radiologic studies evidence signs of fracture consolidation of the subtrochanteric fracture (Figure 7). No signs of infection are also evidenced.

## 3. Discussion

Osteopetrosis is a disease of osteoclasts that results in failure of bone remodeling. Histologically, mature osteopetrotic fracture callus contains no Haversian organization and has a paucity of osteoclasts [5]. Fractures occur frequently in patients with osteopetrosis, and there are several difficulties associated with operative treatment, including the extreme hardness of the bone, which impedes drilling and cutting, hardware failure, periprosthetic fractures, coxa vara deformity, delayed union, pseudoarthrosis, refracture, and periprosthetic infection [2, 6]. The use of blocked plate is the most reported strategy for the management of diaphyseal fractures in patients with osteopetrosis; the said indication is associated with a lower technical difficulty, despite the high nonunion rate presented by these cases [6, 7]. Endomedular nailing in diaphyseal fractures in patients with osteopetrosis presents great technical difficulties but greater mechanical stability in relation to the use of plaque [8]; we consider that the combined use of implants increases fracture focus stability after the first failure of implants. There are fewer reports describing treatment of femoral neck fractures in adults. Armstrong et al. treated seven patients nonoperatively with nonweightbearing, and all had developed coxa vara that subsequently was treated with a valgus osteotomy. In three patients, the fractures were treated with either pins or a compression screw and all united. One patient was treated nonoperatively with nonweightbearing, but a developed nonunion was treated with pins at 6 months, after which the fracture united [2]. Rolauffs et al. reported a 39-year-old man with a femoral neck fracture treated with multiple parallel screws [9]. Three of the four screws broke, the fracture settled into varus, and osteomyelitis of the femur subsequently developed. In our patient, we decided to use a 3.5 mm blocked plate to grant absolute stability to the fracture site, associated with an endomedular nail for the pseudoarthrosis site. There were no signs of nonunion or osteosynthesis failure associated with the femoral neck fracture. The complex management of osteoporotic bone infection is directly associated with its poor bone quality. There is no literary consensus regarding the management of such infections [10]. It was decided to use an endomedular nail coated with PMMA with antibiotic (vancomycin) for the local treatment of the infection associated with intravenous antibiotic treatment prolonged for 6 weeks (vancomycin). In spite of the osteosynthesis failure, there was no recurrence of the infection after such treatment. Park et al. reported good results using blocked plates for the treatment of long bone pseudoarthrosis, using it as an augmentation technique associated with an endomedular nail [11]. However, there are no current reports that refer to the use of this technique for the treatment of infected pseudoarthrosis in the context of a patient with osteopetrosis.

## 4. Conclusion

Despite the difficult management of the patient and the multiple surgeries required, we consider that the use of PMAA-coated endomedular nail and antibiotics, associated with a blocked plate as an augmentation, is a technique to be considered for the management of infected pseudoarthrosis of long bones in patients with osteopetrosis.

## Data Availability

No data availability. A case report.
