# Supplementary material for: Treatment of Infected Pseudoarthrosis in a Subtrochanteric Fracture in a Patient with Osteopetrosis
Source: Case Rep Orthop. 2020 May 4;2020:5630202. doi: 10.1155/2020/5630202 (PMC7232726; doi:10.1155/2020/5630202)
Supplement: Supplementary Materials [file 5630202.f1.pdf]

December 26th, 2019

To whom it may concern:

We are pleased to submit our case report to your Journal, given its high academic standards in trauma and orthopaedic care.

Our article provides reliable information of the difficult treatment of an infected pseudoartrosis in a patient with osteopetrosis. We are well aware of all the limitations of this study; however, given the scarcity of literature on the topic, we believe this manuscript can aid in understanding the possible difficulties in the treatment of this complication in patients with this rare condition.

We certify that all the authors (Lionel Llano, Jorge Barla. Diego Campelo, Danilo Taype, Guido Carabelli, Carlos Sancineto) had contributed to the production of the submitted article and will assume public responsibility for it.

All authors were thoroughly involved in both the drafting and profound revision of the manuscript before submission and have approved the final version to be published. All authors have read the “Guide for authors” guideline and accept the instructions and the conditions in it.

If the case report is accepted it will not be published elsewhere in the same or similar form, in English or in any other language, without written consent of the copyright holder.

Lionel Ezequiel Llano
